# The role of semaphorin 3A on chondrogenic differentiation

**DOI:** 10.1007/s11626-024-00909-z

**Published:** 2024-05-10

**Authors:** Eri Tsuboi, Yuki Asakawa, Naoto Hirose, Makoto Yanoshita, Chikako Sumi, Mami Takano, Azusa Onishi, Sayuri Nishiyama, Naoki Kubo, Daiki Kita, Kotaro Tanimoto

**Affiliations:** Department of Orthodontics and Craniofacial Developmental Biology, Hiroshima University Graduate School of Biomedical and Health Sciences, 1-2-3 Kasumi, Minami-Ku, Hiroshima, 734-8553 Japan

**Keywords:** Semaphorin3A, Cartilage, Endochondral ossification, Cartilage substrate, ATDC5

## Abstract

Osteoblast-derived semaphorin3A (Sema3A) has been reported to be involved in bone protection, and Sema3A knockout mice have been reported to exhibit chondrodysplasia. From these reports, Sema3A is considered to be involved in chondrogenic differentiation and skeletal formation, but there are many unclear points about its function and mechanism in chondrogenic differentiation. This study investigated the pharmacological effects of Sema3A in chondrogenic differentiation. The amount of Sema3A secreted into the culture supernatant was measured using an enzyme-linked immunosorbent assay. The expression of chondrogenic differentiation-related factors, such as Type II collagen (COL2A1), Aggrecan (ACAN), hyaluronan synthase 2 (HAS2), SRY-box transcription factor 9 (Sox9), Runt-related transcription factor 2 (Runx2), and Type X collagen (COL10A1) in ATDC5 cells treated with Sema3A (1,10 and 100 ng/mL) was examined using real-time reverse transcription polymerase chain reaction. Further, to assess the deposition of total glycosaminoglycans during chondrogenic differentiation, ATDC5 cells were stained with Alcian Blue. Moreover, the amount of hyaluronan in the culture supernatant was measured by enzyme-linked immunosorbent assay. The addition of Sema3A to cultured ATDC5 cells increased the expression of Sox9, Runx2, COL2A1, ACAN, HAS2, and COL10A1 during chondrogenic differentiation. Moreover, it enhanced total proteoglycan and hyaluronan synthesis. Further, Sema3A was upregulated in the early stages of chondrogenic differentiation, and its secretion decreased later. Sema3A increases extracellular matrix production and promotes chondrogenic differentiation. To the best of our knowledge, this is the first study to demonstrate the role of Sema3A on chondrogenic differentiation.

## Introduction

The vertical growth of the mandibular bone is mainly dependent on the growth of the mandibular branch, and the growth of the mandibular branch is mainly dependent on “endochondral ossification,” which occurs in the mandibular condyle. Endochondral ossification is the differentiation of undifferentiated mesenchymal cells into cartilage cells after the formation of cartilage primordia, followed by the differentiation of the cartilage cells into hypertrophic cartilage cells and the gradual conversion of the surrounding osteoblasts into limestone. The cartilage tissue is replaced with the bone tissue through ossification (Goldring *et al*. 2006; Mackie *et al*. 2008).

Semaphorin, which we focused on in this study, is a group of proteins identified as repulsive nerve guidance factors that determine the direction of nerve axons (Kolodkin *et al*. 1993). More than 20 types of semaphorin molecules function not only in the nervous system but also in various physiological processes, such as the skeletal, vascular, endocrine, and immune systems have been discovered (Behar *et al*. 1996; Sekido *et al*. 1996; Kumanogoh *et al*. 2002; Serini *et al*. 2003). The semaphorin family has unique structural feature of having a region (sema-domain) consisting of approximately 500 amino acids outside the cell and is classified into eight subgroups based on the difference in the structure on the C-terminal side following the sema domain. In addition, semaphorin is classified into three types depending on its mode of binding to the cell membrane: cell membrane-bound, glycosylphosphatidylinositol bound, and secretory. Among the eight subgroups, class 3 semaphorin is a secretory semaphorin typical of vertebrates, and there are seven members from semaphorin 3A (Sema3A) to semaphorin 3G classified depending on the differences in the base sequence (Yazdani and Terman 2006). Sema3A has a high affinity for Neuropilin-1 (NRP-1), whereas NRP-1 performs intracellular signaling by forming a receptor complex with PlexinA1 (PLXNA1) (Chen *et al*. 1997; Kolodkin *et al*. 1997; Winberg *et al*. 1998; Takahashi and Strittmatter 2001). In addition, in a co-culture experiment of a post-chicken ganglion cell mass and Sema3A, the neurites on the side of Sema3A-expressing cells regressed; therefore, Sema3A was initially identified as a repulsive axonal inducer (Kaneko *et al*. 2006). Recently, osteoblast derived Sema3A has been reported to be involved in bone protection by increasing bone mass by simultaneously promoting osteoblast differentiation and suppressing osteoclast differentiation via estrogen regulation (Hayashi *et al*. 2012, 2019). In addition, Sema3A knockout mice have been reported to exhibit chondrodysplasia such as dyscoupling of costal cartilage or sternum (Behar *et al*. 1996). In addition, the expression of Sema3A and its receptors in the cartilage has been reported (Sumi *et al*. 2018). Thus, Sema3A may play an important role in cartilage growth. Therefore, this study was aimed to elucidate the pharmacological effects of Sema3A on chondrogenic differentiation.

## Materials and methods

### Cell line and culture conditions

All experiments were performed using the mouse chondrogenic cell line ATDC5 (RIKEN Cell Bank, Tsukuba, Japan), which is derived from embryonal carcinoma cells AT805 (Atsumi *et al*. 1990). ATDC5 cells provide an in vitro differentiation model that faithfully replicates the complex differentiation stages of chondrocytes from undifferentiated mesenchymal cells to mineralization under consistent culture conditions (Shukunami *et al*. 1997). Therefore, ATDC5 cells are widely utilized in research concerning the proliferation and differentiation of chondrocytes (Nakatani *et al*. 2007; Yoshioka *et al*. 2015; Yamaguchi *et al*. 2018). Cells were seeded and cultured in 6-well plates (FALCON, Franklin Lakes, NJ) at a density of 6.0 × 10^4^ cells/well. The culture was maintained in Dulbecco’s Modified Eagle’s Medium/Nutrient Mixture F-12 Ham (DMEM/Ham’s F12; Sigma Aldrich, St. Louis, MO) supplemented with 5% fetal bovine serum (FBS; Biological Industries, Cromwell, CT), 10 μg/mL human transferrin (Sigma Aldrich), and 3 × 10 ^−8^ M sodium selenite (Sigma Aldrich) under an atmosphere of 5% CO_2_ in a humidified incubator at 37 °C. The medium was changed every alternate day. When the density of the cells on the plates reached 50% confluence, the cells were changed to a differentiation medium, DMEM/Ham’s F12 containing 5% FBS (Biological Industries, Kibbutz Beit Haemek, Israel), 10 μg/mL bovine insulin (Sigma Aldrich), and 37.5% ascorbic acid 2-phosphatase (Sigma Aldrich) to induce chondrogenic differentiation. The cells were cultured in a differentiation medium for 24 d.

### Measurements of Sema3A protein concentrations in the supernatant of ATDC5 cultures

The cell culture supernatants were collected. Particulates were removed by centrifugation for 15 min at 1000 × *g* and 2–8 °C. The concentrations of Sema3A in the supernatant was measured using a mouse Sema3A enzyme-linked immunosorbent assay (ELISA) kit (Cusabio, Wuhan, China). This assay employs a quantitative sandwich enzyme immunoassay technique, according to the manufacturer’s guidelines. Standard curves were generated using a standard process. The experiments were performed in triplicates.

### Sema3A application

To examine the effect of Sema3A on chondrogenic differentiation, ATDC5 cells were treated with Sema3A (1, 10, and 100 ng/mL; recombinant mouse Sema3A Fc chimera; R&D Systems Inc., Minneapolis, MN) at the time of medium change on the seventh day and the cells are cultured for 24 h. Representative data were obtained from four samples from each group. To investigate the long-term effects of Sema3A on Aggrecan gene expression, 10 ng/ml Sema3A was added continuously by changing the medium every two days, and the cells were cultured for 7 d. On the other hand, to investigate the effects of Sema3A on the synthesis capacity of proteoglycans and the amount of hyaluronan synthesis in the extracellular matrix, 10 ng/ml Sema3A was added continuously during medium change, and the cells were cultured for 13 d.

### Real-time qPCR

Total RNA was isolated from cell cultures using the TRIzol reagent (Invitrogen Life Technologies Inc.) according to the manufacturer’s instructions. cDNA was generated using the ReverTra Ace qPCR RT Master Mix (Toyobo, Osaka, Japan). Real-time qPCR was performed using the THUNDERBIRD SYBR qPCR Mix (Toyobo) and a Light Cycler System (Roche Diagnostics, Mannheim, Germany) to quantify the target gene expression. The primer sets used are listed in Table 1. Relative gene expression levels were calculated using S29 as an internal control. Normalized cycle threshold (Ct) values were compared with those of controls. Data were calculated as a relative expression by 2-ΔCt, where the cycle threshold is the beginning of logarithmic amplification and ΔCt is the difference in the target gene Ct subtracted from the reference gene Ct. A minimum of four independent measurements were obtained.

**Table 1.** The primer sequences for qPCR analysis

Gene	Sequence of primers (5'-3')		Amplicon Size (bp)
*Sema3A*	Forward	CAGCCATGTACAACCCAGTG	154
	Reverse	ACGGTTCCAACATCTGTTCC	
*ACAN*	Forward	AGTGGATCGGTCTGAATGACAGG	105
	Reverse	AGAAGTTGTCAGGCTGGTTTGGA	
*COL2A1*	Forward	CAGGGCTCCAATGATGTAGA	129
	Reverse	CTTCTGTGATCGGTACTCG	
*COL10A1*	Forward	CCTGGTTCATGGGATGTT	137
	Reverse	CTTGTTCTCCTCTTACTGGAATC	
*Sox9*	Forward	AACATGGAGGACGATTGGAG	157
	Reverse	TCCCCTCAAAATGGTAATGAG	
*Runx2*	Forward	CCGCACGACAACCGCACCAT	289
	Reverse	CGCTCCGGCCCACAAATCTC	
*HAS2*	Forward	GATTATGTACAGGTGTGTGAC	76
	Reverse	CCTCTAAGACCTTCACCATC	
*S29*	Forward	ACGGTCTGATCCGCAAATAC	137
	Reverse	CATGATCGGTTCCACTTGGT	

### Alcian blue staining

To assess the deposition of total glycosaminoglycans during chondrogenic differentiation, ATDC5 cells were stained with Alcian Blue. 10 ng/ml of Sema3A was added during medium change every two d and Alcian Blue staining was performed on the 13th day of differentiation. The cells were fixed with a 4% paraformaldehyde solution and stained with 1% Alcian blue staining solution (Sigma) for 1 h at 25 °C. After washing the wells with pure water, plates were photographed. Alcian blue dye was extracted using a 6 M guanidine hydrochloride solution. The absorbance was measured at 450 nm using a microplate reader (Multiskan FC, Thermo Scientific, Waltham, MA).

### Measurement of hyaluronan content in culture supernatant

10 ng/ml Sema3A was added during medium change every two d, and the culture supernatants were collected on days 7, 9, 11, and 13 of differentiation. The concentrations of Hyaluronan in the supernatant were measured using a Hyaluronan quantification kit (Cosmo Bio, Tokyo, Japan). This assay employs a quantitative sandwich enzyme immunoassay technique, according to the manufacturer’s guidelines. Standard curves were generated using a standard process. The experiments were performed in triplicates.

### Statistical analysis

All experiments were repeated at least thrice. Statistical analyses were performed using one-way analysis of variance. Subsequently, Scheffe’s multiple comparison test or Mann–Whitney U test (Statcel 4 software; OMS Publishing Inc., Saitama, Japan) were performed when necessary. A p-value < 0.05 was regarded as indicative of a statistically significant difference. A p-value < 0.01 was regarded as indicative of a highly significant difference.

## Results

### Expression of Sema3A at each differentiation stage of ATDC5 cells

**Figure 1.**
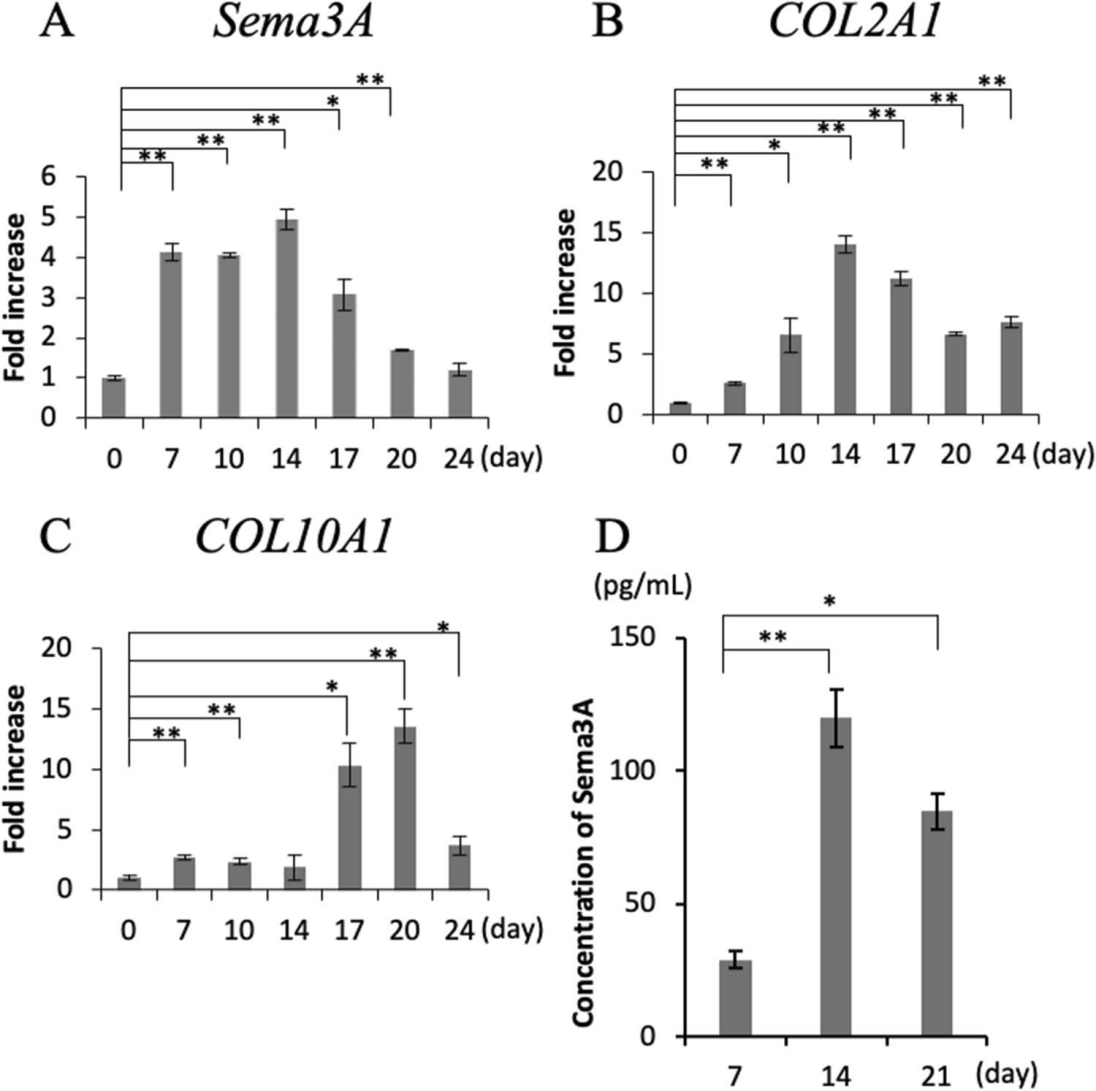
Expression of semaphorin3A(Sema3A) and chondrogenic differentiation markers at each differentiation stage of ATDC5. ATDC5 cells were cultured in differentiation medium for 24 d. The expression levels of (***A***) *Sema3A*, (***B***) *type II collagen* (*COL2A1*), and (***C***) *type X collagen* (*COL10A1*) was determined using qPCR. (*p < 0.05, **p < 0.01, *N* = 4) (***D***) A mouse Sema3A ELISA kit was used to measure the total Sema3A protein content in the supernatant of cultured ATDC5 cells on days 7, 14, and 21. (*p < 0.05, **p < 0.01, *N* = 12).

To confirm the expression of Sema3A at each differentiation stage in ATDC5 cells, we examined the mRNA expression levels using qPCR. *Sema3A* gene expression significantly increased from day 7 after the onset of differentiation, reaching a maximum on day 14, consistent with *Type II collagen* (*COL2A1*) gene expression (Fig. 1*A* and *B*). From day 17, *Sema3A* gene expression decreased, and *Type X collagen* (*COL10A1*) gene expression increased (Fig. 1*A* and *C*). When the amount of Sema3A protein secreted into the culture supernatant of ATDC5 cells was measured using ELISA, it reached a maximum on day 14 of differentiation and decreased on day 21, but both were significantly larger than that on day 7 (Fig. 1*D*).

### Effects of Sema3A on chondrogenic differentiation

**Figure 2.**
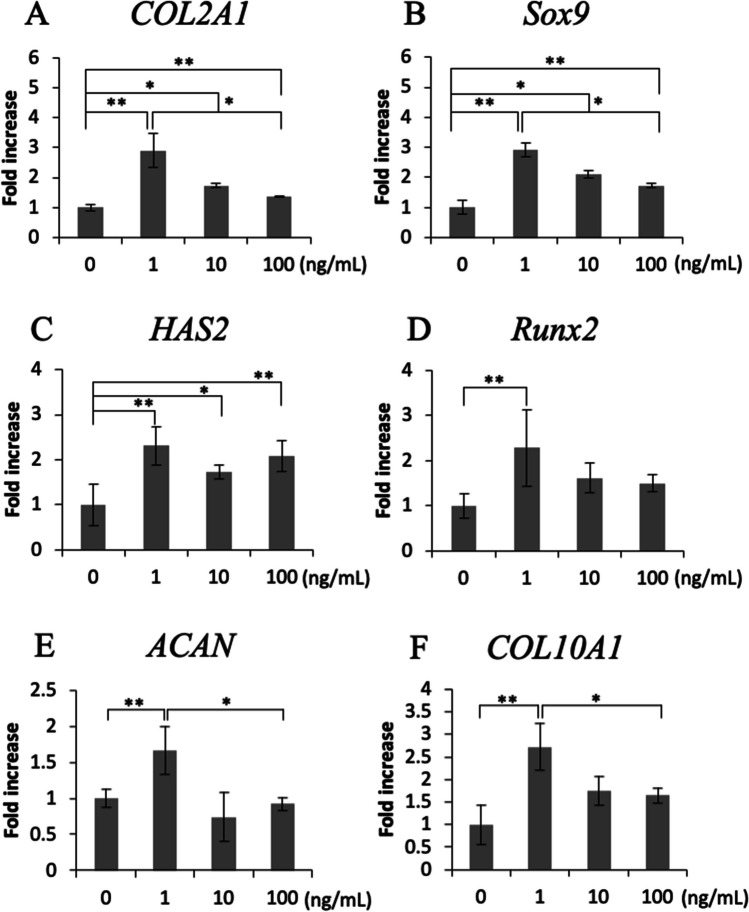
Effects of Sema3A on chondrogenic differentiation. ATDC5 cells were treated with 0, 1, 10, or 100 ng/mL Sema3A for 24 h on day 7. The gene expression levels of (***A***) *COL2A1*, (***B***) *SRY-box transcription factor 9* (*Sox9*), (***C***) *hyaluronan synthase 2* (*HAS2*), (***D***) *Runt-related transcription factor 2* (*Runx2*), (***E***) *Aggrecan* (*ACAN*), and (***F***) *COL10A1* were determined using qPCR. (*p < 0.05, **p < 0.01, *N* = 16).

To investigate the effects of Sema3A on the expression of chondrogenic differentiation markers, we examined its gene expression levels using qPCR. The addition of 1 ng/mL Sema3A to cultured ATDC5 cells on day 7 significantly increased Aggrecan (ACAN), COL2A1, COL10A1, Runt-related transcription factor 2 (Runx2), SRY-box transcription factor 9 (Sox9), and hyaluronan synthase 2 (HAS2) gene expression (Fig. 2*A*‒*F*). Furthermore, the expression of COL2A1, Sox9 and HAS2 was significantly enhanced by the addition of Sema3A at 10 and 100 ng/mL (Fig. 2*A*‒*C*).

### Effect of Sema3A on extracellular matrix synthesis

**Figure 3.**
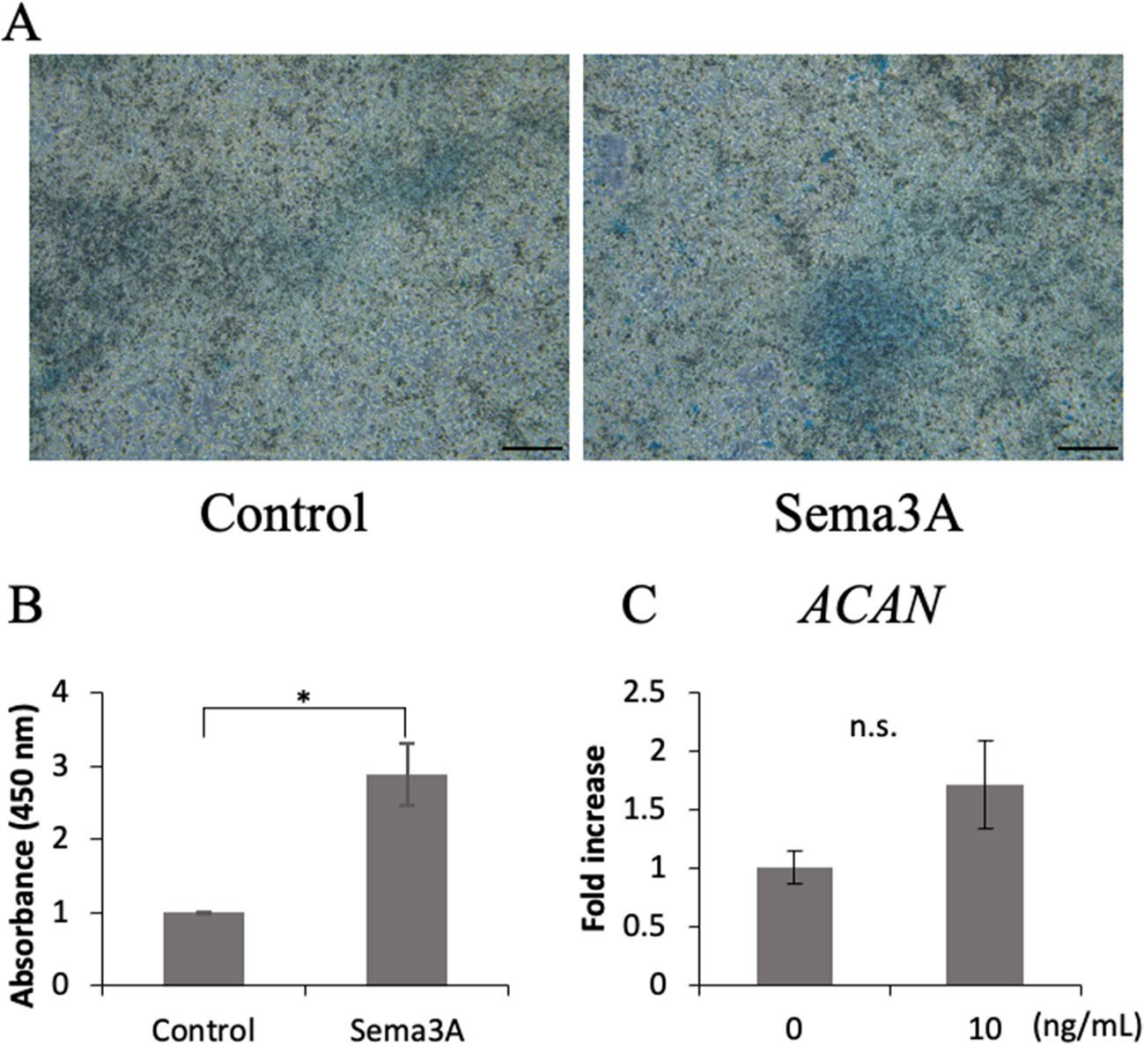
Effects of Sema3A on the ability to synthesize proteoglycans. For Alcian blue staining, Sema3A was added at a concentration of 10 ng/mL for 13 d. (***A***) To assess the deposition of total glycosaminoglycans during chondrogenic differentiation supplemented with Sema3A, ATDC5 cells on day 14 were stained with Alcian blue. (Scale bar: 200 μm) (***B***) Absorbance was measured at 450 nm using a microplate reader. (*p < 0.05, *N* = 8) (***C***) ATDC5 cells were treated with 10 ng/ml Sema3A continuously every two d for 7 d. The gene expression levels of ACAN were determined using qPCR.

**Figure 4.**
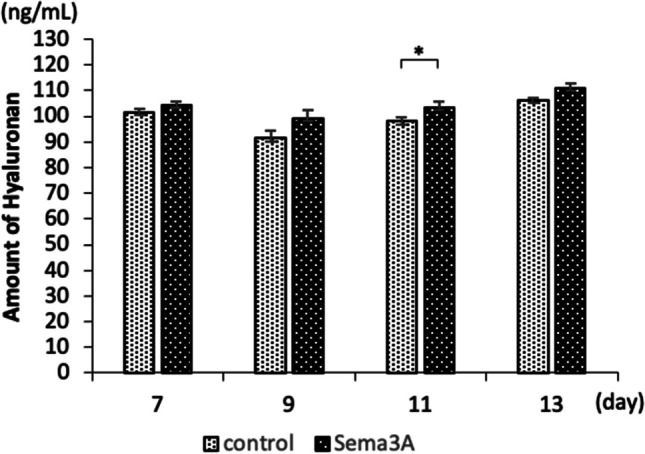
Effects of Sema3A on the ability to synthesize hyaluronan. To evaluate the amount of hyaluronan synthesized during differentiation of ATDC5 supplemented with Sema3A, culture supernatants on days 7, 9, 11, and 13 were collected and the amount of hyaluronan was measured. Negative control (n.c); No statistical difference (n.s.)(*N* = 12).

To evaluate the effect of Sema3A on the ability to synthesize proteoglycans, ATDC5 cells were stained with Alcian blue (Fig. 3*A*). The absorbance of the proteoglycans in the Sema3A group was significantly higher than that in the control group (Fig. 3*B*). Additionally, when 10 ng/ml Sema3A was added continuously every two d for 7 d, an increasing trend in ACAN gene expression was confirmed (Fig. 3*C*). To examine the effect of Sema3A on the amount of hyaluronan that constitutes the extracellular matrix, the amount of hyaluronan was measured. When sema3A was added, the amount of hyaluronan tended to increase (Fig. 4*A*).

## Discussion

In this study, we clarified the role of Sema3A on chondrogenic differentiation. First, we analyzed the gene expression of *Sema3A* in ATDC5 cells. *Sema3A* expression showed a trend consistent with that of *COL2A1*, which significantly increased from day 7 after the onset of differentiation and reached a maximum on day 14. From day 17, *Sema3A* gene expression decreased and *COL10A1* gene expression increased. Sema3A secretion into the culture supernatant peaked on day 14 and decreased by day 21. This trend revealed that Sema3A is abundantly secreted in the early stage of chondrogenic differentiation, and its secretion decreases in the late stages. To the best of our knowledge, this is the first study to clarify Sema3A expression during chondrogenic differentiation.

Next, we examined the effects of excessive Sema3A administration on cartilage differentiation. We added Sema3A on the 7th day of differentiation, where Sema3A secretion capacity was the lowest. The dose of Sema3A in this experiment was determined with reference to the previous studies and the amount of Sema3A protein expressed in the culture supernatant. To investigate the effect of Sema3A on human dental pulp stem cells, Yoshida *et al*. added recombinant Sema3A at a concentration of 10 ng/ml to the culture medium (Yoshida *et al*. 2016). Kajii *et al*. also added Sema3A at a concentration of 1 ng/ml or 100 ng/ml to the culture medium of human chondrocytes to investigate the functions of Sema3A and PLXNA2 in human chondrocytes (Kajii *et al*. 2018). From these reports, the concentration of Sema3A added was determined to be 1, 10, 100 ng/ml. Since the Sema3A protein expression level on day 14, which showed the maximum expression, was about 0.12 ng/ml, the concentrations of Sema3A added in this experiment were 10, 100 and 1000 times the expression level.

In the Sema3A addition experiment, *Sox9* and *Runx2* gene expression was significantly enhanced by the addition of Sema3A on the seventh day. Although the regulation of *Sox9 and Runx2* expression remains unknown, recent studies on ATDC5 have shown that the small G protein Rac1 may be a positive regulator of Sox9-mediated chondrogenesis (Woods *et al*. 2007). When Sema3A binds to NRP-1 and stimulates PLXNA1, FARP2 binds to PLXNA1 immediately below the cell membrane, dissociates, and activates Rac1(Zhou *et al*. 2008). Therefore, Sema3A may regulate Sox9 expression via Rac1. Sox9 has been reported to induce the expression of Aggrecan and Type II collagen (Yonashiro *et al*. 2009). In the present study, the addition of Sema3A enhanced the gene expression of *COL2A1*, suggesting that it was secondarily induced by Sox9, whose expression was enhanced by the addition of Sema3A.

It has been reported that Runx2 expression is low in proliferating chondrocytes and high in pre-hypertrophic chondrocytes, promoting chondrocyte hypertrophy (Nishimura *et al*. 2012). On the other hand, Runx2 maintains the expression of Col2A1 through the intron 6 enhancer and is involved in the regulation of early chondrocyte differentiation markers (Nagata *et al*. 2022). Additionally, Runx2 directly regulates Gpr132, Sfn, c-Myb, and cyclinA1, controlling chondrocyte proliferation (Chen *et al*. 2014). The transcription factor Dmrt2 promotes chondrocyte hypertrophy by binding to Sox9 and Runx2 (Ono *et al*. 2021). From these reports, it can be suggested that Runx2 plays multiple roles in the proliferation, differentiation, and hypertrophy of chondrocytes. From the present research, it is suggested that Sema3A increases not only Sox9 but also Runx2 gene expression, indicating its potential to promote chondrocyte differentiation. COL10A1 expression is regulated by Runx2, and the increased Runx2 by Sema3A may contribute to the promotion of COL10A1 gene expression.

The synthesis and degradation of hyaluronan are regulated according to the chondrocyte differentiation stage, and the expression of hyaluronan synthase HAS2 is upregulated in hypertrophic chondrocytes in the growth plate (Magee *et al*. 2001; Suzuki *et al*. 2005). Sema3A addition increases *HAS2* and ACAN expression. Furthermore, the addition of Sema3A up to day 13 of differentiation significantly increased proteoglycan synthesis, indicating that Sema3A promotes extracellular matrix production.

## Conclusions

In conclusion, Sema3A is secreted during the early stages of chondrogenic differentiation, and its secretion decreases during the late stages of chondrogenic differentiation. Sema3A increased extracellular matrix production and promoted chondrogenic differentiation. This study suggests Sema3A as one of the factors controlling endochondral ossification in the mandibular condyle, and it may be used as a therapeutic method for promoting the growth of the mandible in the future.

## Data Availability

The data that support the findings of this study are available from the corresponding author, Naoto Hirose, upon reasonable request.
